# Schools as a Framework for COVID-19 Epidemiological Surveillance of Children in Catalonia, Spain: A Population-Based Study

**DOI:** 10.3389/fped.2021.754744

**Published:** 2021-09-08

**Authors:** Aida Perramon, Antoni Soriano-Arandes, David Pino, Uxue Lazcano, Cristina Andrés, Martí Català, Anna Gatell, Mireia Carulla, Dolors Canadell, Gemma Ricós, M. Teresa Riera-Bosch, Silvia Burgaya, Olga Salvadó, Javier Cantero, Mònica Vilà, Miriam Poblet, Almudena Sánchez, Anna M. Ristol, Pepe Serrano, Andrés Antón, Clara Prats, Pere Soler-Palacin

**Affiliations:** ^1^Universitat Pompeu Fabra, Barcelona, Spain; ^2^Paediatric Infectious Diseases and Immunodeficiencies Unit, Hospital Universitari Vall d'Hebron, Barcelona, Spain; ^3^Department of Physics, Universitat Politècnica de Catalunya (UPC·BarcelonaTech), Barcelona, Spain; ^4^Agència de Qualitat i Avaluació Sanitàries de Catalunya (AQuAS), Barcelona, Spain; ^5^Respiratory Viruses Unit, Department of Microbiology, Hospital Universitari Vall d'Hebron, Barcelona, Spain; ^6^Comparative Medicine and Bioimage Centre of Catalonia (CMCiB), Fundació Institut d'Investigació en Ciències de la Salut Germans Trias i Pujol (IGTP), Badalona, Spain; ^7^Equip Pediatria Territorial Alt Penedès-Garraf, Barcelona, Spain; ^8^Àrea Bàsica de Salut Pla d'Urgell (Mollerussa), Lleida, Spain; ^9^Centre d'Atenció Primària Barberà del Vallés, Barcelona, Spain; ^10^Centre d'Atenció Primària Drassanes, Barcelona, Spain; ^11^Equip d'Atenció Primària Vic Nord, Barcelona, Spain; ^12^Equip d'Atenció Primària Manlleu, Barcelona, Spain; ^13^Centre d'Atenció Primària Llibertat Reus, Tarragona, Spain; ^14^Corporació del Maresme i la Selva, Barcelona, Spain; ^15^Equip d'Atenció Primària Horta, Barcelona, Spain; ^16^Equip Territorial Pediàtric Sabadell Nord, Barcelona, Spain; ^17^Centre d'Atenció Primària Les Hortes, Barcelona, Spain; ^18^Centre d'Atenció Primària Can Serra Hospitalet de Llobregat, Barcelona, Spain

**Keywords:** COVID-19, SARS-CoV-2, schools, epidemiology, child, tracing, contact

## Abstract

**Objective:** We describe and analyze the childhood (<18 years) COVID-19 incidence in Catalonia, Spain, during the first 36 weeks of the 2020-2021 school-year and to compare it with the incidence in adults.

**Methods:** Data on severe acute respiratory syndrome coronavirus 2 (SARS-CoV-2) tests were obtained from the Catalan Agency for Quality and Health Assessment. Overall, 7,203,663 SARS-CoV-2 tests were performed, of which 491,819 were positive (6.8%). We collected epidemiological data including age-group incidence, diagnostic effort, and positivity rate per 100,000 population to analyze the relative results for these epidemiological characteristics.

**Results:** Despite a great diagnostic effort among children, with a difference of 1,154 tests per 100,000 population in relation to adults, the relative incidence of SARS-CoV-2 for <18 years was slightly lower than for the general population, and it increased with the age of the children. Additionally, positivity of SARS-CoV-2 in children (5.7%) was lower than in adults (7.2%), especially outside vacation periods, when children were attending school (4.9%).

**Conclusions:** A great diagnostic effort, including mass screening and systematic whole-group contact tracing when a positive was detected in the class group, was associated with childhood SARS-CoV-2 incidence and lower positivity rate in the 2020-2021 school year. Schools have been a key tool in epidemiological surveillance rather than being drivers of SARS-CoV-2 incidence in Catalonia, Spain.

## Introduction

On 13^th^ March 2020, the government of Catalonia, Spain, ordered the closure of schools in an effort to contain the spread of severe acute respiratory syndrome coronavirus 2 (SARS-CoV-2) (1). A total of 1,565,478 students from 5,492 Catalonian schools were forced to remain at home during the first pandemic wave, and the schools were not reopened until the beginning of the 2020-2021 academic year on 14^th^ September 2020. Non-pharmaceutical interventions (NPIs), following the protocol launched by the Department of Education of the Catalonian Government (2), were implemented in the educational setting from the beginning of the school year: hand-washing, mandatory mask use for children older than 5, social distancing, organization of children and teachers into bubble groups whenever possible in order to maintain, as far as possible, the same groups of individuals to facilitate contact tracing, and enhanced ventilation in the classroom by keeping doors and windows open, along with other public health practices, such as screening and quarantine the whole group whenever a positive was detected. In fact, it has been demonstrated that all of these measures are essential to mitigate the transmission of SARS-CoV-2 in schools (3–7). However, clusters of SARS-CoV-2 infection have been detected in any number of educational centers. Although educators seemed to play a central role in in-school transmission networks (8), the role of the schools in the SARS-CoV-2 transmission among children (9), and the impact of the school closures on children and their parents (10, 11), remain at the center of public debate worldwide. In fact, closing schools interrupts learning and has a significant negative impact on society as a whole and on children's health and well-being, potentially leading to inequity issues and a loss of years of life (12).

The incidence of SARS-CoV-2 infections in educational settings seems to be a reflection of SARS-CoV-2 transmission at the community level (13, 14). A study performed in Canada comparing school-related cases and outbreaks of COVID-19 to those in the general population detected more than seventy school clusters, but the weekly incidence of SARS-CoV-2 infection in the schools was always lower than the incidence in the whole population, and the authors concluded that schools were not a significant driver of SARS-CoV-2 transmission (15), mainly when compared to households (16). Additionally, a recent study suggests that children in the age-group of 0-9 years do not have substantial rates of SARS-CoV-2 infection during school attendance and are unlikely to play a substantial role in the spread of the infection (17).

Hence, to measure the role of schools in COVID-19 transmission, we examined and analyzed the childhood (<18 years of age) COVID-19 incidence in Catalonia (Spain) during the first 36 weeks of the 2020-2021 school-year (14^th^ September-31^st^ May), including Christmas (22^nd^ December-10^th^ January) and Easter (29^th^ March-5^th^ April) holidays, in comparison to the incidence of COVID-19 in adults.

## Methods

### Data Description

Catalonia is an autonomous region in north-eastern Spain with 7,653,845 inhabitants (1,384,382 under 18 years of age). We obtained public data on the total tested and confirmed SARS-CoV-2 cases in Catalonia, provided by the Catalan Agency for Quality and Health Assessment (AQuAS) that had downloaded the original data from the Catalan Epidemiological Surveillance Network's clinical microbiological laboratories (18). No additional data were available.

A confirmed COVID-19 case was defined as any individual testing SARS-CoV-2 positive by molecular assays [polymerase chain reaction (PCR) or transcription-mediated amplification (TMA)] or rapid antigen testing (RAT) (PANBIO COVID-19 by ABBOTT©) in a respiratory specimen which has a sensitivity of 98.1% (95% CI: 93.2–99.8%) and a specificity of 99.8% (95% CI: 98.6–100.0%) in symptomatic patients, according to the company information. RAT has only been available since 23^rd^ October 2020 (7th week of the school-course) in primary care settings. All the close contacts of the confirmed cases were tested, mainly with PCR. Nevertheless, in the schools, all the classroom contacts were tested by PCR and not by RAT.

This study used aggregated public health data from the health surveillance system to investigate the risk of SARS-CoV-2 among children. No personal or identifiable data were collected; thus, the institutional research board waived the requirement for parental and/or patient informed consent. This information was critical for supporting health-policy recommendations on easing COVID-19 lockdown measures and allowing children to return to school safely. This was identified as a public health priority and as part of the public health response to the COVID-19 pandemic in Catalonia. The results have been used to provide an evidence base for informing national guidance and public health policy to help protect children and staff in educational settings.

### Data Analysis

In Spain, the educational system for those under 18 years has four educational levels. A first non-compulsory stage that corresponds to nursery education (0–5 years) is divided into two cycles, kindergarten (0–3 years) and pre-school (3–5 years). The second stage is the elementary education (6–11 years), which is compulsory, and it is followed by secondary education (12–16 years). The fourth stage is non-compulsory and comprises the high-school education (16–18 years), intermediate vocational training, intermediate professional plastic arts and design courses, and intermediate sports courses (19). According to these stages, and including adults as an independent control group, we focused our research on age-group incidence (cases per 10^5^ population), diagnostic effort (tests per 10^5^ population), and positivity, defined as the quotient between positive SARS-CoV-2 tests (cases) and the total of SARS-CoV-2 tests performed, given as a percentage:

(1) $$\begin{array}{l} \text{positivity }(\%)\;=\text{ number of confirmed cases}/\text{number of}\\ \text{tests performed} \end{array}$$

If an individual was tested at different times for the same clinical episode, delimited by the maximum incubation period for the SARS-CoV-2 infection, we defined the following dates: when all the tests were negative, we considered the date of the first test performed in the clinical episode; and when a test was positive, we relied on the date of that test regardless of the previous negative results in the same episode. Each episode counted once to analyze the dynamics of the epidemic. For each of the variables (i.e., incidence, diagnostic effort, and positivity) and age-groups, we assessed its relative value with regards to that of the general population. These relative variables allow for a rapid identification of those values above the average (<1) and those below (<1).

In addition, we performed a one-way analysis of variance (ANOVA) test to explore differences in the number of the diagnostic tests performed per 10^5^ population, incidence of cases (cases per 10^5^ population), and positivity between the different age-groups. The one-way ANOVA returns the *p*-value for a group of data. It tests the hypothesis that the different samples tested are drawn from populations with the same mean. A *p*-value below 0.05 indicates that the hypothesis is rejected with a 95% significance level.

Holidays and school periods were shared by children of all educational stages until higher education (20). The vacation periods considered for testing were between 21^st^ December 2021 and 10^th^ January 2021 for Christmas, and between 27^th^ March and 5^th^ April 2021 for Easter holidays. To analyze the infections associated with the school-closure periods for holidays, and considering an average period of 7 days between the infection and the diagnosis (approximately 5 days until symptoms onset and 2 days from symptoms onset to the doctor's appointment in Catalonia) (21, 22), the incidence and positivity were defined between 28^th^ December 2020 (i.e., one week after the beginning of the Christmas holidays) and 17^th^ January 2021 (i.e., one week after returning to school). For the Easter holidays this period was defined as between 3^rd^ and 12^th^ April 2021.

### Epidemiological Context

Schools re-opened in Catalonia on 14^th^ September 2020 after a long closure period, starting 13^th^ March 2020. This reopening occurred in a context in which the average 14-day cumulative incidence was around 180–190 cases per 10^5^ population, and the most prevalent SARS-CoV-2 Pangolin lineage was B.1.177 with other minor lineages. To mitigate the risk of transmission, a set of NPIs was established: (1) face masks were mandatory in all educational centers for children older than 5, (2) natural ventilation and hand hygiene in the classrooms and common spaces were recommended, (3) infants and adolescents were clustered into bubble-groups (mainly restricted to classmates) in all educational centers with no interactions with other groups in the center, and (4) whenever a positive case was detected, the whole group was screened and, whatever the result of the SARS-CoV-2 tests, the whole group was quarantined for 10 days. In addition, several mass screening campaigns were carried out in educational centers located in high incidence areas. Furthermore, during the analyzed period, the regional government introduced other community NPIs to reduce the global incidence of COVID-19, as shown in Supplementary Table 1 (23). Eventually, on 9^th^ May 2021, the Spanish Government relaxed some of the previous interventions and allowed mobility throughout the country.

## Results

### Diagnostic Effort

**Figure 1:**
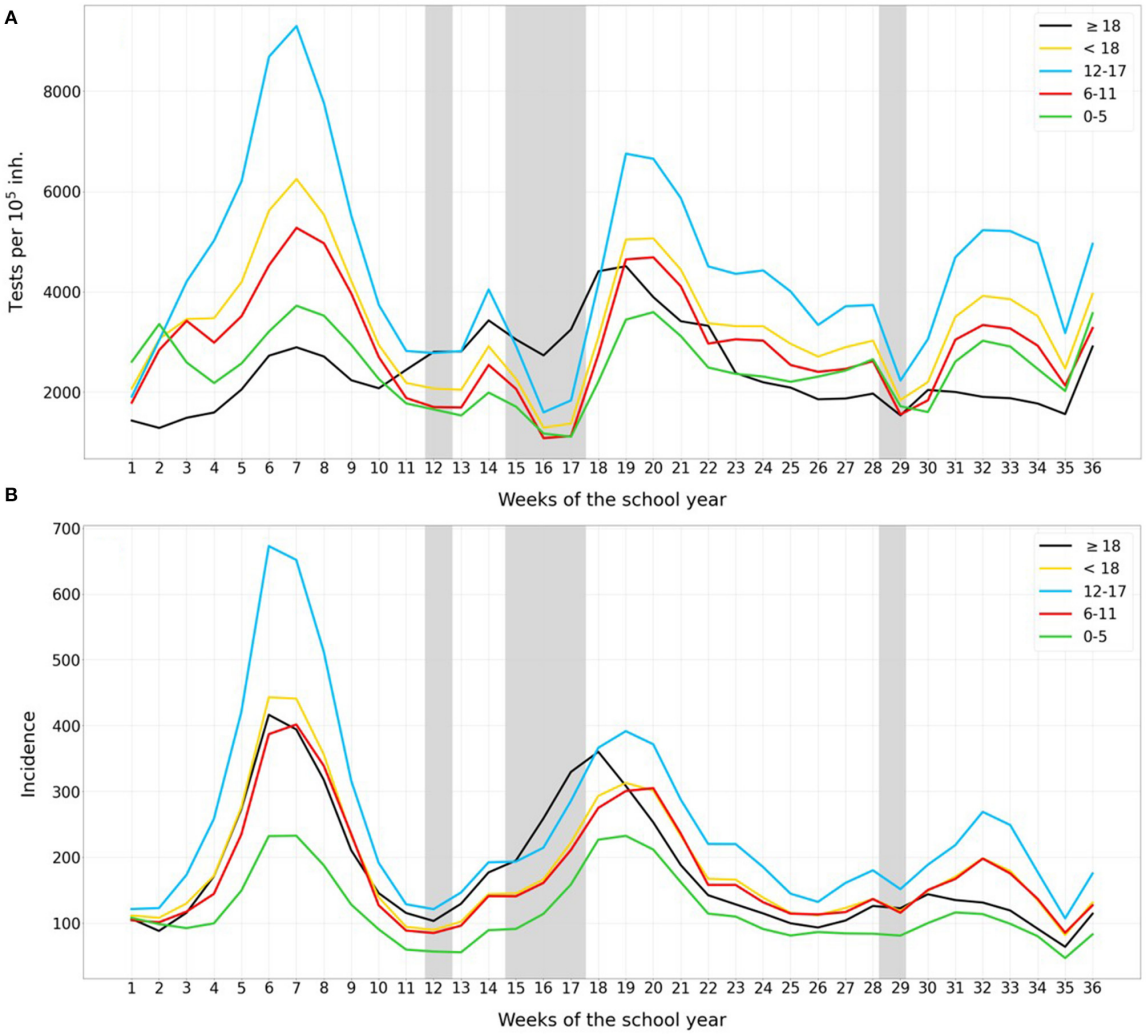
Number of weekly COVID-19 **(A)** tests and **(B)** cases per 10^5^ population, for kindergarten and pre-school children (0–5 years old, green), elementary school children (6–11 years old, red), secondary school children (12–17 years old, blue), adults (black), and the average for all children (yellow). Holiday periods are marked in gray. A 7-day running average was calculated.

During the study period, 1,654,099 SARS-CoV-2 tests were performed in the population <18 years, representing 1.2 tests per person, and 91,857 of them were positive (5.5%). RAT was used for testing in the 34.4% of the 0–5 year-old children, 20.2% of the 6–11 y-old, and 19.6% of 12–17 y-old. Furthermore, 5,554,060 SARS-CoV-2 tests were performed in the adult population, representing less than one test performed per person (0.9), and 400,506 of them were positive (7.2%). The diagnostic effort during the analyzed period was generally greater in children than in adults (yellow and black lines in Figure 1A, respectively), with a difference of 1,335 tests per 10^5^ population between children and adults. For the three different waves of the pandemic occurring since mid-September 2020 (weeks of the school-year: 1–11, 14–24, and 26-35), the mean children/adults test ratios per 10^5^ population per week were: 1.87 (3,910/2,086), 0.97 (3,228/3,326), and 1.63 (2,995/1,839), respectively. However, this diagnostic effort largely varied, especially among children, during the study period (see Figure 1A). Apart from vacation time, the diagnostic effort was clearly greater in all the children's age groups, particularly for the 12–17 years old with a mean of 4,925 tests per 10^5^ population (light blue line of Figure 1A) vs. 2,355 tests per 10^5^ population for adults. Only during 14 weeks of the study period (between week 11 and 23, and during week 30) was the diagnostic effort among the 0-5 years-old lower than for the adults, with a mean of−1,051 tests per 10^5^ population. Four of these 14 weeks were holidays. At Christmas (approximately weeks 15–17), tests were mainly performed on adults, with this the period having the lowest number of tests for children, and yielding a mean difference of−806 tests per 10^5^ population compared to adults.

### Age-Group Incidence

Sars-CoV-2 incidence in children was slightly higher than the incidence in adults for most of the study-period, with a mean difference of 21 cases per 10^5^ population (yellow and black lines in Figure 1B, respectively). This difference was above the mean during the third wave (weeks 30–34), with a difference of 27 cases per 10^5^ population. However, during these weeks, just after the Easter holidays, the diagnostic effort difference between children and adults was higher, as shown in Figure 1A (a mean difference of 1154 tests per 10^5^ population between children and adults in this period). SARS-CoV-2 incidence was directly associated with the age of the children: averages of 248, 175, and 118 cases per 10^5^ population for the group of 12–17, 6–11, and 0–5 years old, respectively, for the study-period. Otherwise, the evolution of incidence for all age-groups followed a similar pattern.

### Relative Diagnostic Effort and Incidence Among the Childhood Age Groups

**Figure 2:**
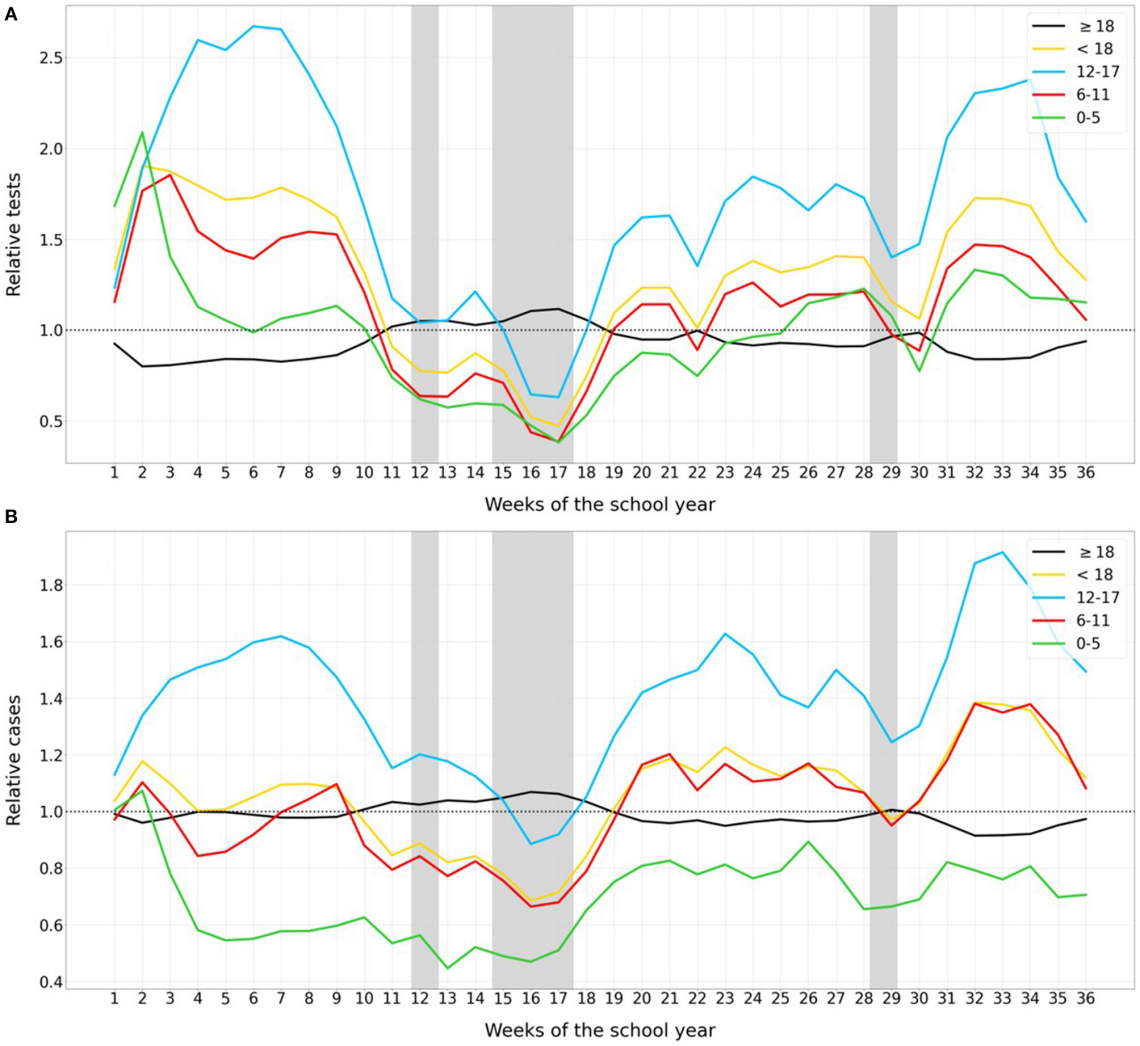
Number of relative weekly COVID-19 **(A)** tests and **(B)** cases in each age group with regards to the general population. Line colors correspond to: kindergarten and pre-school children (0–5, green), elementary school children (6–11, red), secondary school children (12–17, blue), adults (black), and the average for all children (yellow). For each age group, the variables are presented relative to the general population (i.e., value in the age group divided by the value in the general population). Holiday periods are marked with gray bands.

Figure 2 shows that relative values of tests per 10^5^ population among children with respect to the value among the general population were higher compared to the adult relative values during the entire period (mean difference of 0.37), except during weeks 12-18. In fact, relative diagnostic effort among children was almost twice that among adults (means 1.61/0.86) in the first 11 weeks of the school year. Older children (12-17 years) were the most tested age-group (mean of 1.9 relative diagnostic effort) in contrast to pre-school children (1.1), children in elementary school (1.3), and adults (0.9). However, during holiday periods these figures were completely different, with an average of 1.03 relative diagnostic effort in the group of 12–17, compared to 0.62, 0.66, and 1.05 for children aged 0–5, 6–11, and adults, respectively. Regarding the relative SARS-CoV-2 incidence (Figure 2B), this was slightly higher in children than in adults with a mean difference of 0.15, except during the holiday periods (−0.19 relative incidence). Adolescents (12–17 years) had the highest relative incidence throughout the study period, with, on average, 1.5 and 1.1 relative values for the school period and holidays, respectively. Among children of less than 5 years of age, despite the higher relative diagnostic effort, with a mean of 1, incidence was clearly lower than for the general population (on average, 0.69 relative incidence).

### Positivity Rate

**Figure 3:**
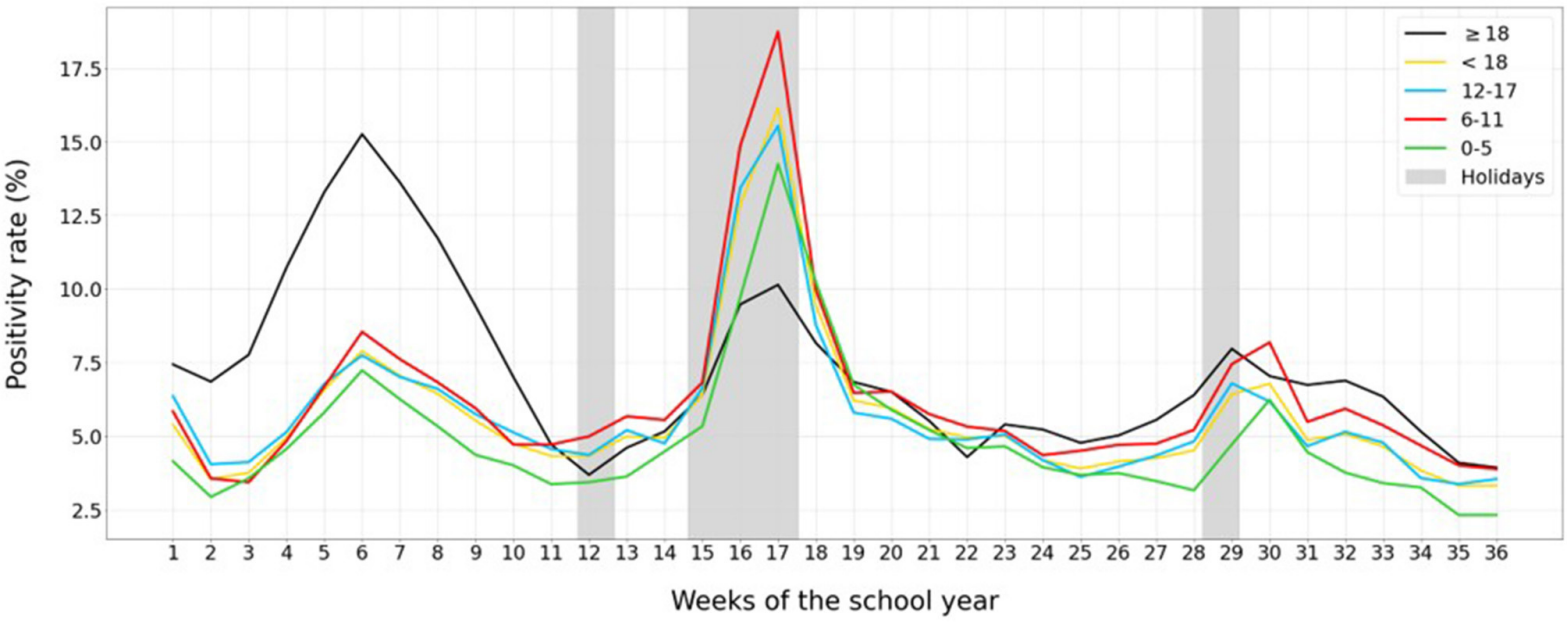
Weekly positivity rate for COVID-19 per 10^5^ population, for kindergarten and pre-school children (0–5, green), elementary school children (6–11, red), secondary school children (12–17, blue), adults (black), and the average for all children (yellow). Holiday periods are marked in gray.

During the first 11 weeks of the school year, positivity among children (yellow line) remained clearly below the positivity among adults (black line), with percentages around 5% or even lower for younger children. This indicates, at least in part, that the higher incidence for the 12–17 years-old age group could be explained by a greater effort at diagnosis. In contrast, after a long weekend (week 12) and during the Christmas holidays (weeks 15-17), the positivity rate for all childhood age groups increased substantially, as shown in Figure 3, because of a lower diagnosis effort (see Figures 1A, 2A).

### Relative Epidemiological Variables Among Different Age Groups Vs. General Population

**Figure 4:**
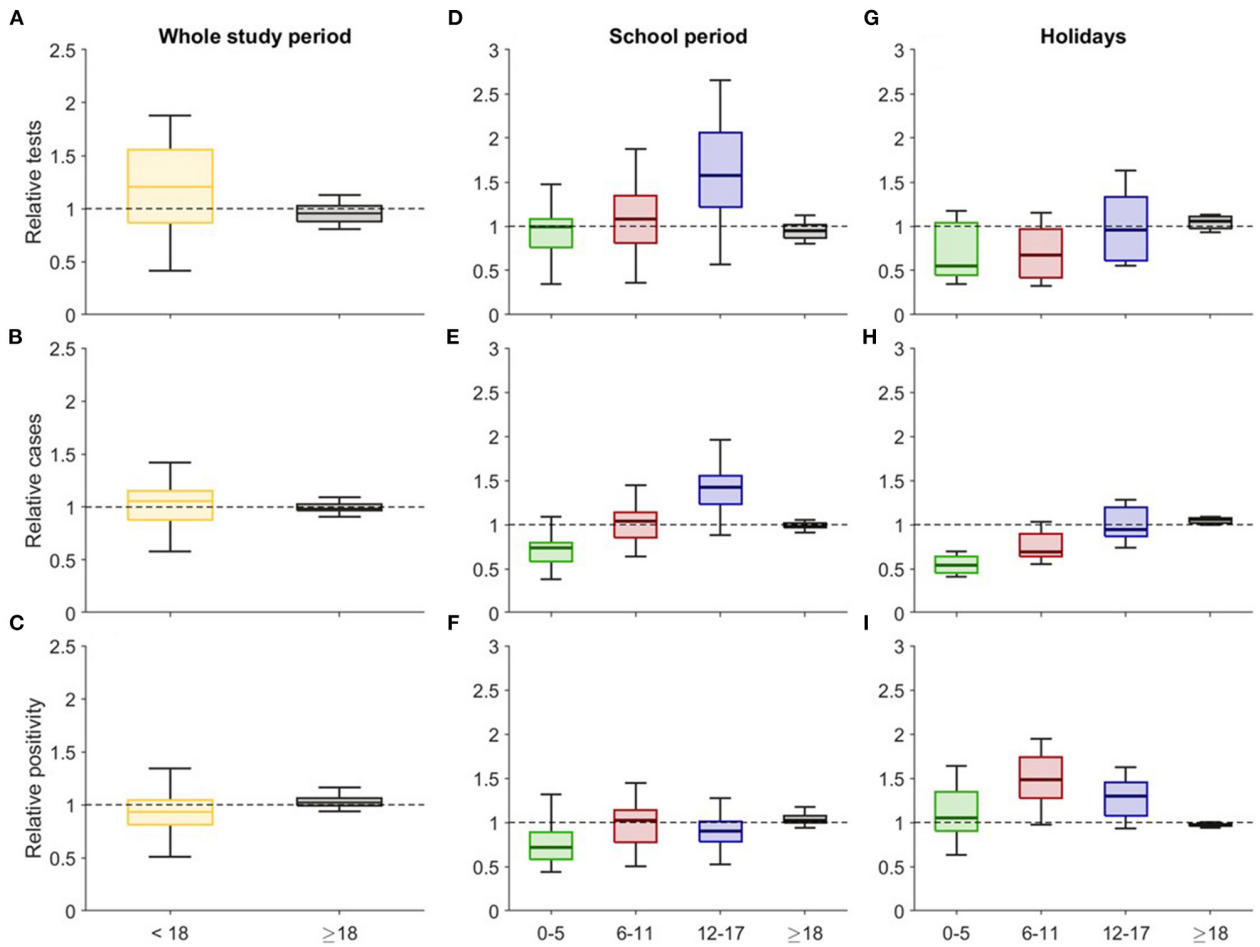
Box plots of the relative daily values of **(A)** number of tests, **(B)** incidence, and **(C)** positivity during the whole study period. Box plots for the relative daily **(D,G)** number of tests, **(E,H)** incidence, and **(F,I)** positivity during the school period and holidays. For each age group, the variables are presented relative to the general population (i.e., value in the age group divided by the value in the general population).

Figure 4 shows the relative diagnostic effort, incidence cases, and positivity among the childhood age groups and adults vs. general population during the entire study period and separating school periods and holidays. Even though the relative number of daily tests among children was significantly higher (*p* < 0.001) than among adults during the study period (Figure 4A, with a mean value of 1.19 and 0.96 for <18 years and adults, respectively), the relative incidence presented similar values, being on average 1.03 for children and 0.99 for adults (Figure 4B). As a consequence, positivity was lower (*p* < 0.001) among children (Figure 4C, with a mean of 0.94 and 1.04 for <18 years and adults, respectively).

We also analyzed separately the school period and the holidays and different age-groups among children. Relative tests decreased considerably for all the children's age groups when the schools were closed (mean values for school period/holidays: 0.97/0.67, 1.09/0.69, 1.62/0.99 for 0–5, 6–11, 12–17 years, respectively), but this was not observed in the adults, who had a similar relative number of tests in the two periods (0.95/1.05, Figures 4D,G). On the other hand, there was a significant difference (*p* < 0.001) between the tests performed within the different age groups during the school period, except for children 0–5 years old when compared to adults (*p* = 0.37). In contrast, during the holidays, there was a significant difference (*p* < 0.001) between the tests performed within the different age groups during the school period, except for children aged 0–5 compared to children 6–11 years old (*p* = 0.83), and for older children (12–17 years) in comparison to adults (*p* = 0.45).

Moreover, the relative incidence among children increased considerably with the age of the cohort in both analyzed periods, being clearly lower than that observed in adults (mean values 0.97 and 1.05 in average during the school period and holidays, respectively) among kindergarten children (0.70 and 0.55, Figures 4E,H). Evidently, during the holidays, the lower number of tests performed substantially decreased the relative incidence among children. Furthermore, it is noteworthy that incidence within different age groups during the school period was significantly different (*p* < 0.005). Nevertheless, performing the same analysis for the holidays, children aged 12–17 showed a non-significant difference when compared to adults (*p* = 0.2).

Finally, relative positivity was smaller for all the children's age groups when compared to the relative positivity among adults during the school period (averages of 0.76, 0.98, 0.91, 1.05 for each age group represented in Figure 4F from left to right), but it was clearly higher among children when the schools were closed (1.09, 1.49, 1.28, 0.97) mainly due to a decrease in the diagnostic effort (Figure 4I). It is of note that, during both the school period and holidays, positivity in all groups differed from each other significantly (*p* < 0.001).

## Discussion

This study shows that, while schools were opened and NPIs were implemented according to the protocol published by the Department of Education of the Catalonian Government (2), COVID-19 incidence among children was similar to that for the adults, but higher among adolescents. However, childhood positivity for SARS-CoV-2 always remained lower due to the greater diagnostic effort, with the systematic testing of whole groups after detection of a positive case and several mass screening campaigns in educational settings. In fact, given the typical mild course of the SARS-CoV-2 infection in children, with a high percentage of asymptomatic cases, the results of this study show the importance of systematic screening in schools whenever a positive case is reported.

The huge increase in SARS-CoV-2 positivity among children during the holidays in comparison with adults is clearly associated with a significant under-diagnosis of pediatric cases during these periods. In contrast, the lower positivity during school periods, with values closer to the objectives of the World Health Organization of 5%, highlights the increase in the diagnostic rate among children biasing their global incidence.

The mean child-adult SARS-CoV-2 incidence ratio was, for the three waves of the pandemic in Catalonia, 1.05, 0.98, and 1.25 (see Supplementary Figure 1 in the Supplementary Material), respectively. However, if the same diagnostic effort were assumed for all the age groups by considering that any positivity rate higher than 5% denotes an under-diagnosis proportional to the distance of the positivity to this baseline (24), the ratio for the three waves would have been 0.61, 1.01 and 0.97, respectively. Thus, the incidence among children was in general lower than for adults and only in the second wave, after the Christmas holidays, was the incidence slightly higher.

Moreover, there are clear differences in the incidence in children attending kindergarten or elementary schools (0–11 years), and adolescents in secondary/high schools (12–17 years) (10, 25). Positivity rates per age group suggest that the former group did not play a significant role in SARS-CoV-2 dissemination in Catalonia probably due to their lower susceptibility to the virus and lesser capacity to transmit it (3). These differences between younger children and teenagers have also been observed elsewhere (9, 10, 26, 27), also recently in our setting (28).

Regarding the influence of Spain's COVID-19 vaccine rollout, adult vaccination started on the 27^th^ of December 2021, and the coverage (full vaccination) went from 0.84% at the end of January 2021 to 17.53% at the end of the study period (see Supplementary Figure 2 in the Supplementary Material). Considering the age <69 years, which is the age-group of active employees, only 5.45% were fully vaccinated by the end of May 2021, while 23.52% had received at least the first dose of a vaccine. Moreover, essential workers, such as teachers and staff working in educational centers, were vaccinated with the Oxford/AstraZeneca© vaccine with a scheduled interval of 12 weeks between the first and the second dose. In particular, in Catalonia, the second dose of the vaccine began to be administered to essential workers on 27^th^ May 2021 (29). Therefore, considering the rollout procedure, the incidence among children during the last part of the analyzed period was probably not affected by the vaccination of teachers and other school workers.

At school week 16, the first SARS-CoV-2 cases related to the B.1.1.7 lineage (Alpha variant) (30) were detected in Catalonia, co-circulating then with the more prevalent B.1.177 lineage. The prevalence of the Alpha variant increased to 17% by week 22 (31). At week 34, the first cases caused by the more transmissible B.1.617.2 lineage (Delta variant) were detected, and the percentage of this variant among randomly selected positive samples reached 10–20 % in the last week of this study period (30^th^ May 2021) (32). Since the novel SARS-CoV-2 variants seem to be characterized by greater transmissibility and have become the most prevalent viruses (11), schools are a perfect scenario for monitoring the pandemic among children and key to initiating contact-tracing studies among students' families. In this sense, these studies should yield improved understanding of the rates of secondary cases after a child is found positive in a class. We did not detect any significant difference in the last weeks of the study period regarding the presence of these more transmissible genetic variants with the incidence and positivity of SARS-CoV-2. However, relative incidence was much higher in adolescents than in previous weeks, but this fact was directly associated with the greater diagnostic effort in the last weeks of the analyzed period, as shown in Figure 2.

Our study has some notable strengths. Firstly, this is a population-based study with data provided by the AQuAS, which downloaded them from the reference Catalan Epidemiological Surveillance Network of all the clinical microbiological laboratories of the region. Secondly, we combined three epidemiological characteristics to improve interpretation of the evolution of the childhood COVID-19 pandemic during the school year, and to collect not only the symptomatic cases but also to be able to detect the asymptomatic children with SARS-CoV-2 infection diagnosed through systematic whole-group testing after detection of a positive case, reinforced with several mass screening campaigns in the schools. And finally, we analyzed our results according to the school stages and holiday periods to permit us to differentiate the epidemiological characteristics for different scenarios both in the school and at home. In fact, this analysis could help to improve public health policies in the future of the pandemic.

However, our study has some limitations. Firstly, we do not know the percentage of asymptomatic children in the study period. Nevertheless, we found a correlation (Supplementary Figure 3) between diagnostic effort and positivity because of the testing protocols in the schools: the greater the diagnostic effort, the lower the positivity. Secondly, we do not know the exact percentage of students and teachers who strictly followed the NPIs at each educational center. Thirdly, we cannot disaggregate the effect of NPI measures implemented in the community which could affect the dynamics of adults and children in a different manner and that were not constant throughout the course. Nevertheless, the epidemiological differences between children and adults in scholar vs. holiday periods reported in this study are expected to be mainly caused by whether the schools were open or not in these two periods. Fourthly, as described before, the percentage of younger children (under 5) tested by RAT was higher than in older age-groups. This is likely because they were mostly tested out of the school contact tracing studies, and therefore incidence in this age-group could be underestimated. Finally, childhood SARS-CoV-2 incidence during the school year has not been demonstrated to be a determining factor in the levels of community transmission, but mitigation measures, mainly NPIs applied at the educational centers, could have been crucial to obtaining such a favorable outcome. Studies of the seroprevalence of IgG against SARS-CoV-2 in school personnel and students have been published, together with information from their partners, to determine whether the prevention measures applied in the centers were effective in controlling transmission. These studies did not detect SARS-CoV-2 infections in children attending day-care during the onset of the second wave of the pandemic (5). The same conclusions were obtained in other studies carried out in Luxembourg (13) and in the United States (4), in which prevention measures were used to prevent transmission in educational centers. In fact, outbreaks of COVID-19 in educational settings seem rare, and the incidence of infections in these centers reflects that of the general population at the community level (33). Another example of a multicenter seroprevalence study conducted in schools, in France, also demonstrated that SARS-CoV-2 seropositive children were more likely than seronegative children to have been exposed to a laboratory-confirmed COVID-19 living adult, suggesting that intrafamily transmission was more plausible than transmission within daycare centers (34).

However, the appearance of new genetic variants of SARS-CoV-2, such as the alpha variant which was firstly detected in the United Kingdom, has shown that they have greater transmissibility in all age groups, but without being associated with higher mortality (30). In a recent article, three childhood education centers with SARS-CoV-2 variant B.1.1.7 and related domestic outbreaks were investigated. Despite the creation of bubble groups to control transmission, the cases occurred in almost all groups, and also among people without close contact. The secondary attack rates for children were similar to those for adults (35).

In conclusion, this study shows that due to the screening campaigns and NPI measures implemented, schools have been a key tool in epidemiological surveillance, and not drivers of the SARS-CoV-2 incidence, in Catalonia, Spain. In addition, they have been a crucial element for the detection of cases in an age-group population with a higher percentage of asymptomatic cases.

## Data Availability Statement

The raw data supporting the conclusions of this article will be made available by the authors, without undue reservation.

## Ethics Statement

Ethical review and approval was not required for the study on human participants in accordance with the local legislation and institutional requirements. Written informed consent from the participants' legal guardian/next of kin was not required to participate in this study in accordance with the national legislation and the institutional requirements.

## Author Contributions

AS-A and CP: conceptualization. MC, AP, and DP: methodology. AA, CP, PS-P, and AS-A: validation. MC, AP, DP, AA, CP, PS-P, and AS-A: formal analysis. UL: resources. AP, DP, CP, and AS-A: writing—original draft preparation. AP and MC: visualization. DP, CP, and AS-A: supervision. All authors writing—review and editing, revised, and approved the final version of the manuscript.

## Conflict of Interest

CP received funding from the Catalan Agency for Quality and Health Assessment AQuAS under the agreement 2021_021OE while the study was being carried out. The remaining authors declare that the research was conducted in the absence of any commercial or financial relationships that could be construed as a potential conflict of interest.

## Publisher's Note

All claims expressed in this article are solely those of the authors and do not necessarily represent those of their affiliated organizations, or those of the publisher, the editors and the reviewers. Any product that may be evaluated in this article, or claim that may be made by its manufacturer, is not guaranteed or endorsed by the publisher.

## Supplementary Material

Supplementary Figure S1 Evolution of the reported (black) and corrected by the positivity rate values (red) ratio of cases of COVID-19 per 100,000 population in children over those in adults.

Supplementary Figure S2 Evolution of the percentage of population vaccinated with 1 (left) and 2 dosis (right) for different age groups during the analyzed period.

Supplementary Figure S3 Correlation matrix for the data frame containing the diagnostic effort, incidence and positivity rates of children and adults during the study period.
