# Supplementary figures and images for: Schools as a Framework for COVID-19 Epidemiological Surveillance of Children in Catalonia, Spain: A Population-Based Study
Source: Front Pediatr. 2021 Sep 8;9:754744. doi: 10.3389/fped.2021.754744 (PMC8457047; doi:10.3389/fped.2021.754744)

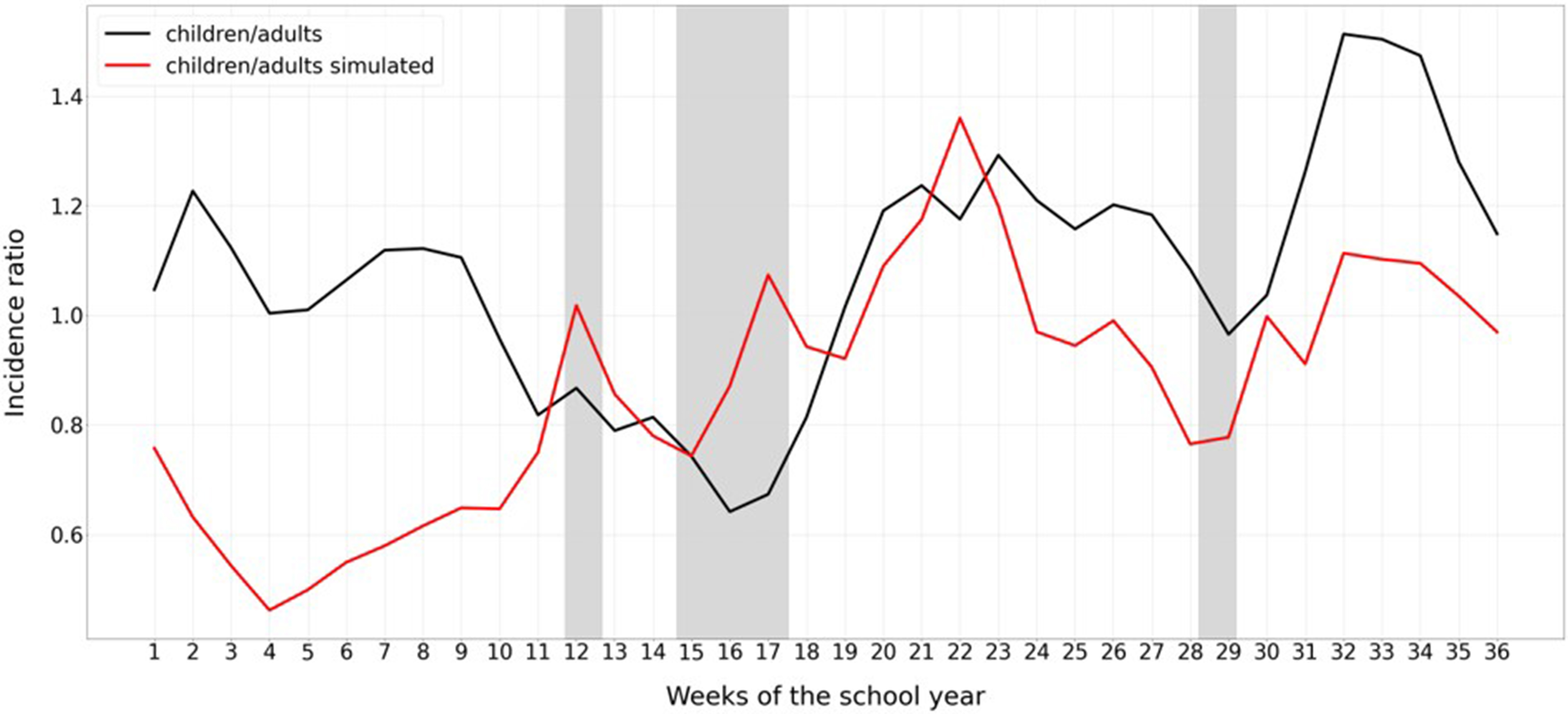

Supplement: Supplementary Figure S1 — Evolution of the reported (black) and corrected by the positivity rate values (red) ratio of cases of COVID-19 per 100,000 population in children over those in adults. [file Image_1.JPEG]

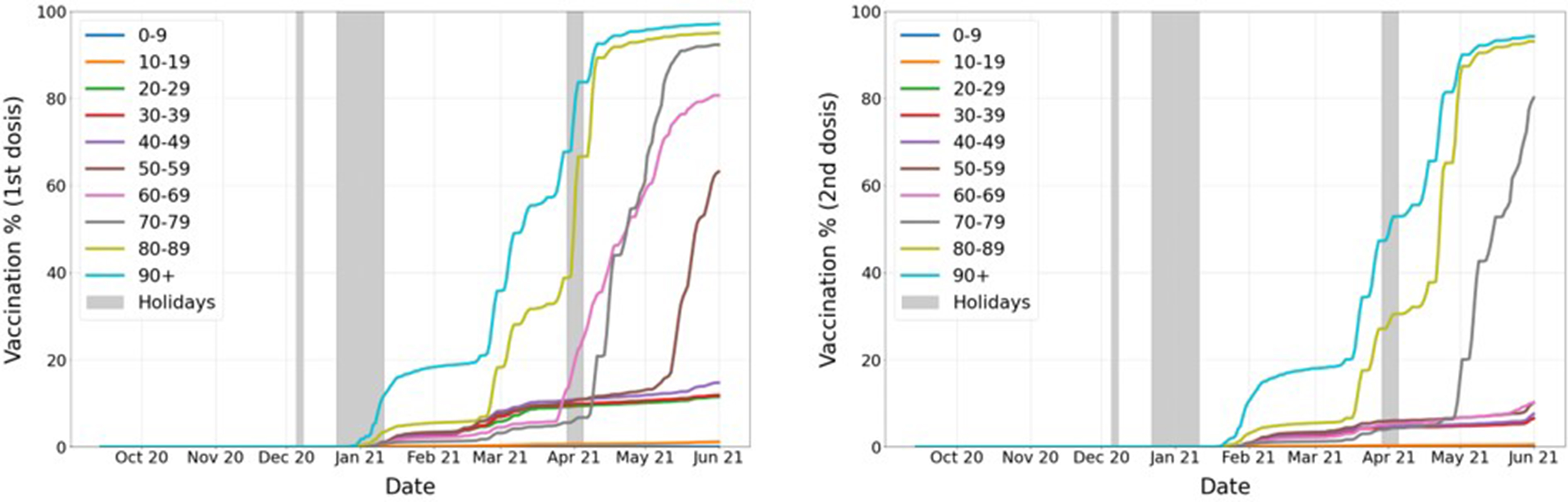

Supplement: Supplementary Figure S2 — Evolution of the percentage of population vaccinated with 1 (left) and 2 dosis (right) for different age groups during the analyzed period. [file Image_2.JPEG]

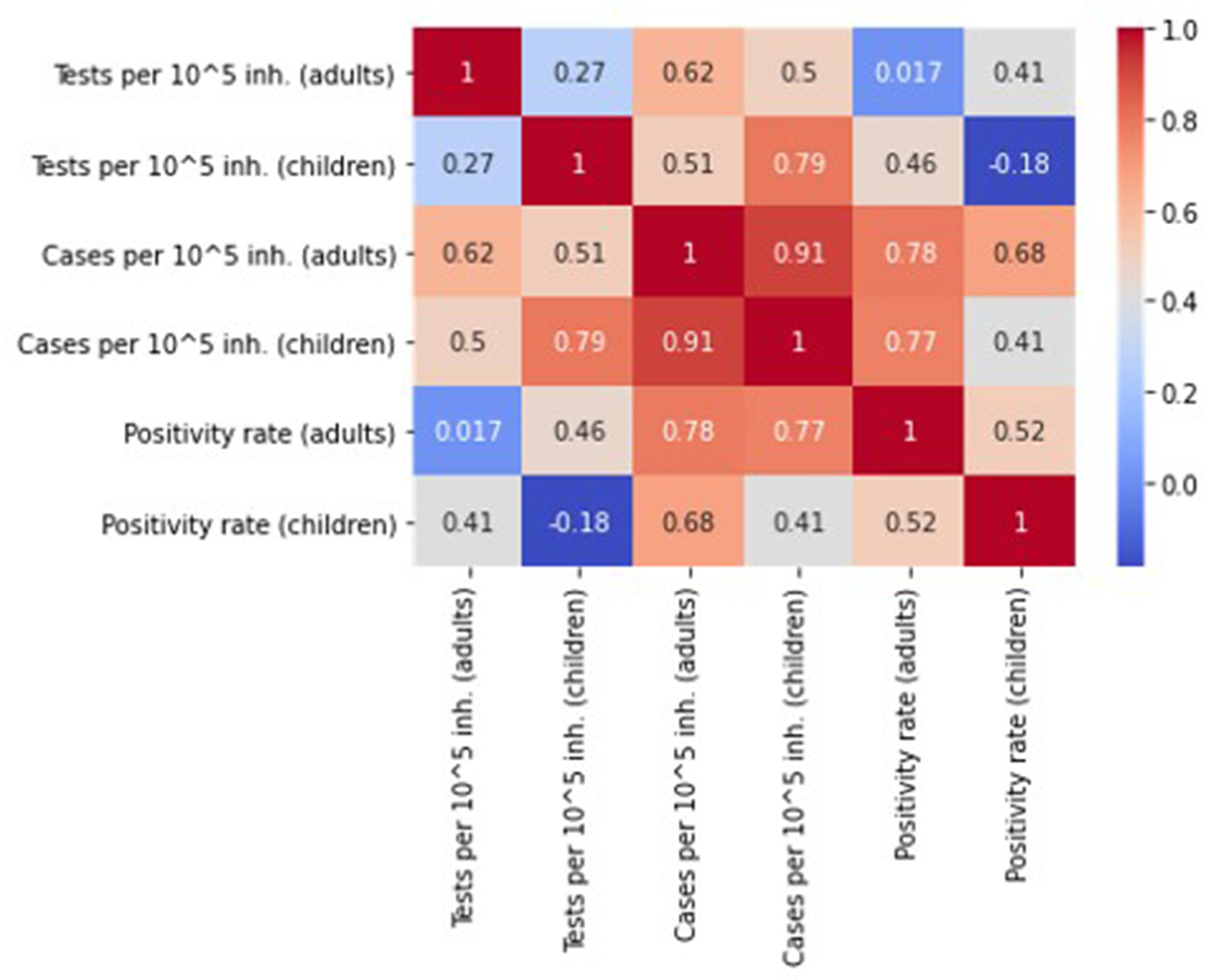

Supplement: Supplementary Figure S3 — Correlation matrix for the data frame containing the diagnostic effort, incidence and positivity rates of children and adults during the study period. [file Image_3.JPEG]
