# Supplementary material for: Schools as a Framework for COVID-19 Epidemiological Surveillance of Children in Catalonia, Spain: A Population-Based Study
Source: Front Pediatr. 2021 Sep 8;9:754744. doi: 10.3389/fped.2021.754744 (PMC8457047; doi:10.3389/fped.2021.754744)
Supplement: Supplementary file 4 [file Table_1.DOCX]

**Table S.1.** Set of NPI measures that the Catalonian government introduced within the 14^th^ of September and the 31^st^ of May.

| Description | Date of application | Details |
| --- | --- | --- |
| 1^st^ set of NPI | 15-10-2020 | - Social activities:   - Meetings of < 6 people   - Between “cohabitation bubbles” and “extended bubbles”   - Limited non-essential travels - Cultural and leisure activities:   - Closed: bingos, casinos, arcades, children's indoor play areas, local festivals   - 50% capacity in cultural activities and preassigned seats   - Closing time at 23:00 h   - Children’s playgrounds open until 20:00 h - Shops and shopping centres:   - 30% capacity in retail businesses   - 1,5 m of minimum distance between customers   - Closed: Beauty centres, except from hairdressers   - 30% capacity in non-sedentary markets - Religious acts and civil ceremonies:   - 50% capacity - Sports:   - 50% capacity in gyms   - Closed: unsupervised sports venues   - Postponed: non-professional competitions   - Not postponed: professional competitions - Hotels and restaurants:   - Closed: only home delivery or pick-up at the establishment by appointment   - 50% capacity in common areas in hotels - Universities and schools:   - Opened: schools, institutes, extracurricular activities and sports   - Universities: online theoretical teaching - Companies:   - Promotion of telework   - Air renovation before and after working hours   - Suspended: congresses, conventions and trade fairs |
| Introduction of RAT | 23-10-2020 | - Introduction of RAT in addition to PCR tests |
| 2^nd^ set of NPI | 25-10-2020 | - Night-time confinement, from 22:00 h to 6:00 h   - Closed to the public at 21:00 h: services, retail businesses, restaurants, sportive and recreational activities and public spaces   - Closed to the public at 22:00 h: cultural activities. Attendees are allowed to return home between 22:00 h and 23:00 h - Exceptions:   - Emergency healthcare   - Acquisition of pharmaceutical products as a matter of urgency   - Travel to and from work, with the corresponding company certificate   - Travel of professionals, and accredited volunteer staff, to provide essential, health and social services   - Care for the elderly, minors, dependents either disabled or especially vulnerable, for undelayable reasons   - Urgent action in the courts   - Return to the place of usual residence   - Pet urgent care from 4:00 h to 6:00 h, individual travel   - Other causes of justified need |
| 3^rd^ set of NPI | 29-10-2020 | - Forbidden to enter or leave Catalonia - During the weekends, forbidden to enter or leave municipality from 6:00 h on Friday until 6:00 h on Monday. Exceptions:   - Visits to the region’s cemeteries on October 31^st^ and All Saints’ Day   - Health services, work trips or causes of justified need   - Individual sports activities between neighbouring municipalities - Closure of bars and restaurants extended. Food delivery at home until 23:00 h - Only loan service in libraries - Closed: theatres, cinemas, concert halls, sports centres and gyms.   - Shopping centres, except for shops that are food and essential services   - Shops of more than 800 m^2^ if the area is not reduced to 800 m^2^ and limit the capacity to 30%   - Extracurricular activities, except those done at school keeping the “group bubble” |
| Relaxation of previous NPI | 23-11-2020 | - Social activities:   - Meetings of < 6 people, within the “usual bubble” - Cultural and leisure activities:   - Closed: bingos, casinos, arcades, children's indoor play areas, local festivals   - 50% capacity in museums, libraries and exposition halls   - 50% capacity and 500 people limit in cinemas, theatres, auditoriums and concert halls   - Children’s playgrounds open until 20:00 h - Shops and shopping centres:   - Closed: shopping centres, except from first need shops with public access   - Opened: establishments offering close physical contact services, including hairdressers and beauty centres, with prior appointment and preventive measures   - 30% capacity in shops and non-sedentary markets - Religious acts:   - 30% capacity - Sports:   - 50% capacity in outdoor sports facilities and equipment   - 30% capacity in indoor facilities, with prior appointments and no dressing rooms, except for swimming activities - Restaurants:   - Home delivery until 23:00 h or pick-up at the establishment until 22:00 h   - Terraces open from 6:00 h to 21:30 h   - 30% capacity in indoor spaces from 6:00 h to 21:30 h   - 4 people per table and 2 m of separation between tables - Extracurricular activities:   - Opened: non-competitive sport activities in school, leisure educational activities outdoors   - Maximum of 6 people in indoor leisure educational activities and on-site extracurricular activities - Education:   - Opened: schools and institutes   - Reduction of on-site activities: baccalaureate, training courses and regulated general education courses   - Universities: online theoretical teaching - Companies:   - Promotion of telework   - Suspended: congresses, conventions and fairs - Lockdown:   - Forbidden to enter or leave Catalunya without justified motivation   - Forbidden to enter or leave the municipality from Friday at 6:00 h to Monday at 6:00 h   - Night-time confinement, from 22:00 h to 6:00 h, except from justified needs |
| December vaccination | 27-12-2020 | - First stage of vaccination |
| January NPI | 07-01-2021 | - Social activities:   - Meetings of < 6 people, from two “cohabitation bubbles”   - Indoor meetings limited to visits to dependent or vulnerable people within the “cohabitation bubble” - Cultural and leisure activities:   - Closed: bingos, casinos, arcades, children's indoor play areas, local festivals   - 50% capacity in cinemas, theatres, auditoriums, concert halls, museums, libraries and exposition halls. 500 people limit indoors, 1000 people limit outdoors   - 50 % capacity in children’s playgrounds, opened until 20:00 h - Shops and shopping centres:   - Closed: shopping centres and shops of more than 400 m^2^, except first need establishments   - 30% capacity in retail businesses, shopping centres of less of 400 m^2^ and non-sedentary markets. Opened from 6:00 h to 21:00 h - Religious acts:   - 30% capacity, maximum of 500 people indoors, 1000 people outdoors - Sports:   - 50% capacity in outdoor sports facilities and equipment, < 6 people   - Closed: indoor facilities and activities, competitions - Restaurants:   - Home delivery until 23:00 h or pick-up at the establishment until 22:00 h   - Terraces open from 7:30 h to 09:30 h and from 13:00 h to 15:30 h   - 30% capacity in indoor spaces from 7:30 h to 09:30 h and from 13:00 h to 15:30 h   - 4 people per table and 2 m of separation between tables - Extracurricular and leisure activities:   - Opened: activities in school or organised by educational centres, within the “educational group bubble”   - Closed: all others - Education:   - Opened: schools and institutes   - Reduction of on-site activities: baccalaureate, training courses and regulated general education courses   - Universities: online theoretical teaching - Companies:   - Promotion of telework   - Suspended: congresses, conventions and fairs - Lockdown:   - Forbidden to enter or leave Catalunya without justified motivation   - Forbidden to enter or leave the municipality without justified reason or to work   - Night-time confinement, from 22:00 h to 6:00 h, except from justified needs |
| February NPI | 08-02-2021 | - Social activities:   - Meetings of < 6 people, from two “cohabitation bubbles”   - Indoor meetings limited to visits to dependent or vulnerable people within the “cohabitation bubble” - Cultural and leisure activities:   - Closed: bingos, casinos, arcades, children's indoor play areas, local festivals   - 50% capacity in cinemas, theatres, auditoriums, concert halls, museums, libraries and exposition halls. 500 people limit indoors, 1000 people limit outdoors   - 50 % capacity in children’s playgrounds, opened until 20:00 h - Shops and shopping centres:   - Closed: shopping centres and shops of more than 400 m^2^, except first need establishments   - 30% capacity in retail businesses, shopping centres of less of 400 m^2^ and non-sedentary markets. Opened from 6:00 h to 21:00 h - Religious acts:   - 30% capacity, maximum of 500 people indoors, 1000 people outdoors - Sports:   - 50% capacity in outdoor sports facilities and equipment, < 6 people   - 30% capacity in indoor facilities and activities, <6 people and wearing masks   - Competitions only in state categories - Restaurants:   - Home delivery until 23:00 h or pick-up at the establishment until 22:00 h   - Terraces open from 7:30 h to 10:30 h and from 13:00 h to 16:30 h   - 30% capacity in indoor spaces from 7:30 h to 10:30 h and from 13:00 h to 16:30 h   - 4 people per table and 2 m of separation between tables - Extracurricular and leisure activities:   - All activities re-opened, groups of <6 people or those maintaining the bubble group. - Education:   - Opened: schools and institutes   - Reduction of on-site activities: baccalaureate, training courses and regulated general education courses   - Universities: online theoretical teaching - Companies:   - Promotion of telework   - Suspended: congresses, conventions and fairs - Lockdown:   - Forbidden to enter or leave Catalunya without justified motivation   - Forbidden to enter or leave the comarca without justified reason or to work   - Night-time confinement, from 22:00 h to 6:00 h, except from justified needs |
| February vaccination | 08-02-2021 | - Second stage of the vaccination |
| March NPI | 01-03-2021 | - Social activities:   - Meetings of < 6 people, from two “cohabitation bubbles”   - Indoor meetings limited to visits to dependent or vulnerable people within the “cohabitation bubble” - Cultural and leisure activities:   - Closed: bingos, casinos, arcades, children's indoor play areas, local festivals   - 50% capacity in cinemas, theatres, auditoriums, concert halls, museums, libraries and exposition halls. 500 people limit indoors, 1000 people limit outdoors   - 50 % capacity in children’s playgrounds, opened until 20:00 h - Shops and shopping centres:   - Closed: shopping centres and shops of more than 400 m^2^, except first need establishments   - 30% capacity in retail businesses, shopping centres of less of 400 m^2^ and non-sedentary markets. Opened from 6:00 h to 21:00 h - Religious acts:   - 30% capacity, maximum of 500 people indoors, 1000 people outdoors - Sports:   - 50% capacity in pools, outdoor sports facilities and equipment   - 30% capacity in indoor facilities and activities, <6 people and wearing masks   - Competitions only in international and state categories - Restaurants:   - Home delivery until 23:00 h or pick-up at the establishment until 22:00 h   - Terraces open from 7:30 h to 10:30 h and from 13:00 h to 16:30 h   - 30% capacity in indoor spaces from 7:30 h to 10:30 h and from 13:00 h to 16:30 h   - 4 people per table and 2 m of separation between tables   **From the 8 of March on, restaurants open from 07:30h to 17:00h.**   - Extracurricular and leisure activities:   - All activities re-opened, groups of <6 people or those maintaining the bubble group.   - Scholar trips allowed. - Education:   - Opened: schools and institutes   - Reduction of on-site activities: baccalaureate, training courses and regulated general education courses   - Universities: online theoretical teaching - Companies:   - Promotion of telework   - Suspended: congresses, conventions and fairs - Lockdown:   - Forbidden to enter or leave Catalunya without justified motivation   - Forbidden to enter or leave the comarca without justified reason or to work   - Night-time confinement, from 22:00 h to 6:00 h, except from justified needs |
|  | 15-03-2021 | - Social activities:   - Meetings of < 6 people   - Indoor meetings limited to visits to dependent or vulnerable people within the “cohabitation bubble” - Cultural and leisure activities:   - 30% capacity in bingos, casinos, arcades, children's indoor play areas, local festivals   - 50% capacity in cinemas, theatres, auditoriums, concert halls, museums, libraries and exposition halls. 500 people limit indoors, 1000 people limit outdoors   - 50 % capacity in children’s playgrounds, opened until 20:00 h - Shops and shopping centres:   - Closed: shopping centres and shops of more than 400 m^2^, except first need establishments   - 30% capacity in retail businesses, shopping centres of less of 400 m^2^ and non-sedentary markets. Opened from 6:00 h to 21:00 h - Religious acts:   - 30% capacity, maximum of 500 people indoors, 1000 people outdoors - Sports:   - 50% capacity in pools, outdoor sports facilities and equipment   - 30% capacity in indoor facilities and activities, <6 people and wearing masks   - All competitions allowed. Professional football and basketball without public. In others, 50% public outdoors and 30% public indoors - Restaurants:   - Home delivery until 23:00 h or pick-up at the establishment until 22:00 h   - Terraces open from 7:30 h to 17:00 h   - 30% capacity in indoor spaces from 7:30 h to 17:00 h   - 4 people per table and 2 m of separation between tables - Extracurricular and leisure activities:   - All activities re-opened, groups of <6 people or those maintaining the bubble group.   - Scholar trips allowed. - Education:   - Opened: schools and institutes   - Reduction of on-site activities: baccalaureate, training courses and regulated general education courses   - Universities: 30% of on-site teaching - Companies:   - Promotion of telework   - Suspended: congresses, conventions and fairs - Lockdown:   - Forbidden to enter or leave Catalunya without justified motivation   - Mobility limited to people within the same “cohabitation bubble”   - Night-time confinement, from 22:00 h to 6:00 h, except from justified needs |
| April NPI | 09-04-2021 | - Social activities:   - Meetings of < 6 people   - Indoor meetings limited to visits to dependent or vulnerable people within the “cohabitation bubble” - Cultural and leisure activities:   - 30% capacity in bingos, casinos, arcades, children's indoor play areas, local festivals   - 50% capacity in cinemas, theatres, auditoriums, concert halls, museums, libraries and exposition halls. 500 people limit indoors, 1000 people limit outdoors   - 50 % capacity in children’s playgrounds, opened until 20:00 h - Shops and shopping centres:   - 30% capacity. Opened from 6:00 h to 21:00 h, including weekends - Religious acts:   - 30% capacity, maximum of 500 people indoors, 1000 people outdoors - Sports:   - 50% capacity in pools, outdoor sports facilities and equipment   - 30% capacity in indoor facilities and activities, <6 people and wearing masks   - All competitions allowed. Professional football and basketball without public. In others, 50% public outdoors and 30% public indoors - Restaurants:   - Home delivery until 23:00 h or pick-up at the establishment until 22:00 h   - Terraces open from 7:30 h to 17:00 h   - 30% capacity in indoor spaces from 7:30 h to 17:00 h   - 4 people per table and 2 m of separation between tables - Extracurricular and leisure activities:   - All activities re-opened, groups of <6 people or those maintaining the bubble group.   - Scholar trips allowed. - Education:   - Opened: schools and institutes   - Reduction of on-site activities: baccalaureate, training courses and regulated general education courses   - Universities: 30% of on-site teaching - Companies:   - Promotion of telework   - Suspended: congresses, conventions and fairs - Lockdown:   - Forbidden to enter or leave Catalunya without justified motivation   - Forbidden to enter or leave the comarca without justified reason or to work   - Night-time confinement, from 22:00 h to 6:00 h, except from justified needs   **From the 26-04-2021 mobility within the whole Catalonia allowed** |
| April vaccination | 01-04-2021 | - Third stage of the vaccination |
| May NPI | 03-05-2021 | - Social activities:   - Meetings of < 6 people   - Indoor meetings limited to visits to dependent or vulnerable people within the “cohabitation bubble” - Cultural and leisure activities:   - 30% capacity in bingos, casinos, arcades, children's indoor play areas, local festivals   - 50% capacity in cinemas, theatres, auditoriums, concert halls, museums, libraries and exposition halls. 500 people limit indoors, 1000 people limit outdoors   - 50 % capacity in children’s playgrounds - Shops and shopping centres:   - 30% capacity. Opened from 6:00 h to 21:00 h, including weekends - Religious acts:   - 30% capacity, maximum of 500 people indoors, 1000 people outdoors - Sports:   - 50% capacity   - All competitions allowed. Professional football and basketball without public. In others, 50% public outdoors and 30% public indoors - Restaurants:   - Home delivery until 23:00 h or pick-up at the establishment until 22:00 h   - Terraces open from 7:30 h to 17:00 h   - 30% capacity in indoor spaces from 7:30 h to 17:00 h   - 4 people per table and 2 m of separation between tables - Extracurricular and leisure activities:   - All activities re-opened, groups of <6 people or those maintaining the bubble group.   - Scholar trips allowed. - Education:   - Opened: schools and institutes   - Reduction of on-site activities: baccalaureate, training courses and regulated general education courses   - Universities: 30% of on-site teaching - Companies:   - Promotion of telework   - Suspended: congresses, conventions and fairs - Lockdown:   - Forbidden to enter or leave Catalunya without justified motivation   - Night-time confinement, from 22:00 h to 6:00 h, except from justified needs |
|  | 09-05-2021  De-escalation | - Mobility allowed throughout Spain - Social activities:   - Meetings of < 6 people   - Indoor meetings restricted and limited visits to dependent or vulnerable people within the “cohabitation bubble” - Cultural and leisure activities:   - 30% capacity in bingos, casinos, arcades, children's indoor play areas, local festivals and amusement parks   - 50% capacity in cinemas, theatres, auditoriums, concert halls, museums, libraries and exposition halls. 500 people limit indoors, 1000 people limit outdoors   - 50 % capacity in children’s playgrounds - Shops and shopping centres:   - Opened until 22:00 h   - Opened from 6:00h to 23:00h, convenience stores - Religious acts:   - 50% capacity, maximum of 500 people indoors, 1000 people outdoors - Sports:   - 50% capacity   - All competitions allowed. Professional football and basketball without public. In others, 50% public - Restaurants:   - Terraces open from 7:30 h to 23:00 h   - 30% capacity in indoor spaces from 7:30 h to 23:00 h   - 4 people per table and 2 m of separation between tables - Extracurricular and leisure activities:   - All activities re-opened, groups of <6 people or those maintaining the bubble group.   - Scholar trips allowed. - Education:   - Opened: schools and institutes   - Reduction of on-site activities: training courses and regulated general education courses   - Universities: 30% of on-site teaching - Companies:   - Promotion of telework   - Suspended: congresses, conventions and fairs. Except for previous authorization |
